# Entomopathogenic Fungi Associated with Exotic Invasive Insect Pests in Northeastern Forests of the USA

**DOI:** 10.3390/insects4040631

**Published:** 2013-11-05

**Authors:** Vladimir Gouli, Svetlana Gouli, José A. P. Marcelino, Margaret Skinner, Bruce L. Parker

**Affiliations:** 1 Entomology Research Laboratory, University of Vermont, 661 Spear Street, Burlington, VT 05405, USA; E-Mails: vgouli@uvm.edu (V.G.); sgouli@uvm.edu (S.G.); mskinner@uvm.edu (M.S.); bparker@uvm.edu (B.L.P.); 2 Azorean Biodiversity Group (GBA, CITA-A) and Portuguese Platform for Enhancing Ecological Research and Sustainability (PEERS), University of the Azores, 9500-321, Portugal

**Keywords:** entomopathogenic fungi, *Myriangium duriaei*, *Hirsutella lecaniicola*, *Beauveria bassiana*, *Colletotrichum fioriniae*, *Lecanicillium muscarium*, *Paecilomyces marquandii*, *Metarhiziopsis microspora*, *piercing-sucking insects*, *Fiorinia externa*, *Parthenolecanium corni*, *Adelges tsugae*, *Taeniothrips inconsequens*

## Abstract

Mycopathogens of economically important exotic invasive insects in forests of northeastern USA have been the subject of research at the Entomology Research Laboratory, University of Vermont, for the last 20 years. Elongate hemlock scale, European fruit lecanium, hemlock woolly adelgid and pear thrips were analyzed for the presence of mycopathogens, in order to consider the potential for managing these pests with biological control. Fungal cultures isolated from insects with signs of fungal infection were identified based on morphological characters and DNA profiling. Mycopathogens recovered from infected insects were subdivided into three groups, *i.e.*, specialized entomopathogenic; facultative entomopathogens; ubiquitous opportunistic contaminants*.* Epizootics were caused by fungi in the specialized group with the exception of *M. microspora*, *P. marquandii* and *I. farinosa*. Inoculation of insects in laboratory and field conditions with *B. bassiana*, *L. muscarium* and *Myriangium* sp. caused insect mortality of 45 to 95%. Although pest populations in the field seemed severely compromised after treatment, the remnant populations re-established themselves after the winter. Although capable of inducing high mortality, a single localized aerial application of a soil-dwelling fungus does not maintain long-time suppression of pests. However, it can halt their range expansion and maintain populations below the economic threshold level without the use of expensive insecticides which have a negative impact on the environment.

## 1. Introduction

Globalization is expanding the established geographical and historical range of many living organisms. Unpremeditated migration of exotic species to North America started with the first settlers from Europe and Asia. Numerous species of microorganisms, plants and animals have adapted to the different climatic zones of Canada, USA and Mexico. As a rule, exotic species create the most severe economic problems because local biota are not adapted to counteract these new immigrants. Agricultural, forest and ornamental plants in North America are severely damaged by many exotic phytophagous species. 

Species with piercing and sucking mouthparts, especially armored scales, adelgids and thrips, have a significant economic impact. In the northeastern region of the USA, widespread defoliation of sugar maple trees periodically occurs as a result of the activity of the exotic pear thrips, *Taeniothrips inconsequens* (Uzel) [1]. This pest was initially identified causing damage to maples in Pennsylvania in 1979, and Vermont in 1985 [2]. In 1988, feeding damage caused by pear thrips contributed to defoliation of over 200,000 hectares in Vermont [3], 400,000 in Pennsylvania [1] and 81,000 hectares in Massachusetts [4]. Other northeastern states, including New York, Connecticut and New Hampshire, were also affected [5]. The complex of exotic invasive species, including hemlock woolly adelgid (HWA), *Adelges tsugae* Annand (Hemiptera: Adelgidae); and elongate hemlock scale (EHS), *Fiorinia externa* Ferris (Hemiptera: Diaspididae), threaten the very existence of hemlock trees. Hemlock woolly adelgid has become the principal pest of eastern hemlock, *Tsuga canadiensis* and Carolina hemlock, *T. carolineana.* It may also become a significant pest of other coniferous trees [6]. The European fruit lecanium scale (LS), *Parthenolecanium corni* Bouche (Hemiptera: Coccidae), also intermittently causes significant damage to a wide variety of forest trees and other agricultural and ornamental plants. The morphological and biological characteristics of thrips, adelgids and scales, together with their high reproductive potential and cold-resistance, facilitate their successful colonization and establishment in vast areas of North America. Application of broad spectrum chemical pesticides is not advised for forests, because of the negative environmental implications, and their inability to provide long term solutions to pest problems. However, the urgency of plant protection often requires their use. The development of alternative strategies to chemical pesticides is urgent. Research activity on alternative management strategies for hemlock woolly adelgid and other exotic invasives has increased since the mid-1990s [7]. Several entomophagous insects including *Laricobius nigrinus*, *Scymnus camptodromus*, *S. ningshanensis*, *S. sinuanodulus*, and *Sasajiscymnus tsugae* (Coleoptera: Coccinellidae) were discovered in China and Japan and have been imported as possible biological control agents against hemlock woolly adelgid in the USA, and some have been successfully established. *Scymnus tsugae*, which was released in Connecticut and Virginia in 1997, reduced hemlock woolly adelgid densities by 47%–83% [8]. 

Another biological control strategy against invasive insect pests is the use of entomopathogenic fungi. Special attention has been given to entomopathogenic fungal research because they have the ability to penetrate through the insect’s cuticle. A significant research initiative began in the 1990s in Vermont directed towards development of entomopathogenic fungi for exotic insects impacting forest health, including pear thrips, hemlock woolly adelgid, elongate hemlock scale, and European fruit lecanium scale. Numerous fungal isolates including *Lecanicillium lecanii*, *Hirsutella* spp., *Beauveria bassiana*, *Metarhizium anisopliae*, *Mariannaea* sp. and others have been recovered from infected pear thrips [9,10]. Other fungal isolates have been found to be associated with elongate hemlock scale and lecanium scale [11,12,13,14]. These entomopathogenic agents offer an opportunity for biological regulation of exotic invasive species, especially when the fungal pathogens are found associated with epizootic processes, where a rapid decline of the pest population is occurring [15]. The humid climate of the northeastern USA favors manifestation of fungal epizootics such as the one observed among populations of gypsy moth, *Lymantria dispar*, which is caused by the entomophthoralean fungus *Entomophthora maimaiga* [16]*.* In May 2006, the Vermont Department of Forests, Parks and Recreation issued a warning that forest tent caterpillar, *Malacosoma disstria*, was forecasted to cause widespread defoliation of 165,000 hectares of forest land., However, in a short period of time, an epizootic caused by the fungus, *Furia gastropachae*, led to the rapid decline of populations and halted the development of the predicted scenario of deforestation. Explosive epizootics are often connected with the activity of entomophthoralean fungi, namely in arthropods with piercing and sucking mouthparts, especially mites [17,18,19] and aphids [20,21,22]. 

Currently thousands of fungal species and strains have been isolated and identified from a vast number of arthropod species around the globe [23]. However, our knowledge of the activity of fungi in natural insect populations and the possibility to apply them for pest control management strategies is limited. This article discusses empirical research on the potential of entomopathogenic and entomophilous fungi as biocontrol agents within populations of exotic invasive insects of the northeastern forests of the USA In addition, it provides standardized sampling protocols, as well as *in vitro* and *in situ* fungal isolation & insecticidal activity protocols. Illustrations of the most common signs and symptoms of disease, found associated with insect epizootics in forest stands of the northeastern USA, are reported. These standardized protocols and illustrations can prove to be an expedient tool to isolate and identify epizootic pathogens *in situ* and *in vitro*. The standardized methodologies reported here were developed by scientists and students at the Entomology Research Laboratory, University of Vermont over the last 20 years.

## 2. Experiment

### 2.1. Standardized Insect Sampling Protocols

Pathogens of forest insects were collected from populations of elongate hemlock scale, European fruit lecanium scale, hemlock woolly adelgid, and pear thrips over the last 20 years of research at the Entomology Research Laboratory (ERL, Burlington, VT, USA)*.*

Symptomatic insects were collected from Vermont, New Hampshire, Massachusetts, New York, and Rhode Island. Pathological material was also collected during routine monitoring activities and numerous surveys in forest stands. Insects with signs of mycoses were also obtained from collectors at universities, forest specialists and members of the general public. In addition, in order to sample for entomopathogenic fungi of hemlock woolly adelgid and elongate hemlock scale, the ERL established six forest sites, located in New Hampshire, Massachusetts, Rhode Island and New York, for the long term monitoring of pathogens of insect pests (1997–2013). These sites were selected where mass insect mortality was observed. In each of the six sample sites, 10 co-dominant eastern hemlock trees with symptomatic insects were selected at random (total 60 trees). As a rule, all surveys were carried on twig samples of approximately 10 cm long with hemlock woolly adelgid and/or elongate hemlock scale were clipped from each tree and processed within 48 hours of collection to isolate fungi. Periodically, biological material from northeastern states was obtained via the United States Department of Agriculture (USDA) Forest Service personnel.

For collection of entomopathogenic fungi from pear thrips—an insect that spends 10 month/year in the soil—samples were collected on sites located in three geographically distinct areas, corresponding to three USDA plant cold hardiness zones (3, 4, and 5). Within each area, two forest stands predominating in sugar maple were identified (each ~10 hectares) and long term sites were established. These were selected because sugar maple trees (*Acer* spp.), exploited for maple syrup production, represent an important economic activity in some New England states, and their protection is of particular interest. Samples were taken over time (1999–2012) in two types of Vermont forests, those with 90% and those with 75% sugar maple trees in the overstory. This permitted an assessment of fungal biodiversity within different forest types over time. The standard methodology for these surveys, within each maple stand, consisted of four plots randomly established 100–200 m apart. At each randomly established plot center, five nearby dominant sugar maple trees were selected. At each tree one soil sample was taken 0.5 m from the bole and one a few cm inside the drip line of the tree using a standard tulip bulb planter. Samples were collected between September and November before the soil froze. Each soil sample was placed in a cylindrical container (10 × 13 cm) and the top covered with a piece of 4-mil transparent plastic sheet, coated on one side with a thin film of Tanglefoot^®^ to catch thrips as they emerged from the soil. Containers with soil samples were kept at room temperature (22 °C ± 2 °C) for 35 days, after which the sticky lids were removed and inspected at 40×. Pear thrips and other arthropods were removed and used for pathological analyses and subsequent isolation of fungi.

Entomopathogenic fungi were also isolated from heavy infestation of European lecanium scale sampled from maple saplings (2007–2010). The standard methodology for these surveys and fungal isolation protocols consisted of five sugar maple trees, selected at random, and from each tree, five twigs, approximately 20–30 cm long and infested with lecanium scale were collected and placed in separate plastic bags. Twigs were inspected microscopically, and scales with obvious signs of fungal infection (*i.e.*, fungal mycelia covering partially or completely the body of the insect) were removed and handled as described below in order to retrieve fungal mycoses.

### 2.2. Pathological Analyses and Isolation of Fungi Protocols

Diseased insects or fresh cadavers were used to prepare slides. Two methods were used: (a) insects were placed individually on slides in a mixture of glycerol and saline solution (1:1 volume) [24] and covered with a cover slip; (b) scotch tape imprint method. The tape method was as follows: insects were placed between two pieces of tape and gently squashed. The two tapes were separated, and each was placed in a droplet of cotton blue stain to detect fungal propagules or other microorganisms in the insect’s body [25]. We found this method allowed for efficient discernment of microorganisms in and on the insect host surface.

Slides were examined using light and phase contrast microcopy, to accurately detect morphological peculiarities of insect tissues and the presence of fungal propagules. Pathological structures were documented by preparation of micrographs.

Two protocols were commonly used over the years to isolate fungi from infected insects. In a first protocol, insects were surface sterilized by dipping them for 1 min. in a solution of 2.5% sodium hypochlorite with 0.08% Silvet L-77^®^ (polysiloxane polyether copolymer), then rinsing them twice with sterile distilled water (SDW) and plating them on three different growth media, *i.e.*, potato dextrose agar (PDA) supplemented with penicillin (0.02 g/L) and streptomycin (0.04 g/L), Sabouraud dextrose agar and yeast (SDAY), and an experimental composition based on medium for cultivation of insect cells (TC-100, Sigma, St. Louis, MT, USA) with the addition of raw egg yolk (10%). For the second protocol, diseased insects or cadavers were homogenized in two ml sterile distilled water (SDW), after surface sterilization, and then the homogenate mixture was diluted 1:10 with SDW. Initial and diluted homogenate mixtures were used for inoculation of the three different media described above. Inoculated Petri dishes were incubated for 3 weeks at 24 °C. Single fungal colonies were transferred to new Petri dishes each week. Isolates were purified using standard dilution techniques [26].

### 2.3. Protocols for the Identification of Fungi

Fungal cultures were identified based on morphological characteristics and DNA analyses. Common saprophytic species were excluded from further research. These included species from the genera *Penicillium*, *Cladosporium*, *Mucor* and some others. Identification based on morphological and cultivation characteristics was done using taxonomic guidelines and fungal fruiting bodies [27,28,29,30]. Fungal specimens were also sent to specialists for verification of identification when necessary. Some isolates were then identified based on DNA analyses following methodologies described in Marcelino *et al*. [13]. Genes commonly used for phylogenetic analysis at the generic level and above (*i.e.*, 28S ribosomal DNA) were selected, as well as genes for within-species differentiation (*i.e.*, the internal transcribed spacers, ITS).

### 2.4. Protocols for the Estimation of Insecticidal Activity of Fungi (*in Vitro* and *in Situ*).

Several of the isolates collected over the last 20 years were assessed for pathogenicity aganist hemlock woolly adelgid and elongate hemlock scale under laboratory and field conditions. Hemlock twigs (15 cm long) infested with hemlock woolly adelgid and/or elongate hemlock scale (50 target insects/branches) were collected in the field. Standard protocols for insecticidal activity of fungi were as follows: first, each twig was put in a glass tube with wet sand to prevent dessication. Subsequently, the twigs were sprayed with a suspension of fungal conidia at 5 × 10^7^ conidia/mL suspended in sterile distilled water (SDW) with 0.02% Silwet (Momentive, Columbus, OH, USA). When a complex of fungi was isolated from diseased or dead insects (e.g., *Myriangium duriaei* & *Colletotrichum fioriniae*; *Lacanicillum lecanii* & *Beauveria bassiana*), these were both suspended in 5 × 10^7^ conidia/mL concentrations in one single solution. A 0.02% solution of Silwet with SDW was used for blank control treatments. The twigs were held vertically in a rack and individually sprayed with a hand-held atomizer at a distance of 30 cm with 200 μL of the treatment solution. Twigs were individually placed in sterile graduated 50 mL conical tubes containing 16 g of sterilized sand and 7 mL sterile distilled water. Each tube was partially covered with a cap to allow ventilation. The tubes were placed in a plastic bag to maintain controlled environmental conditions (22 °C with 16:8 LD). The twigs were inspected after 3, 5 and 7 days to determine the number of live and dead insects, from which the percent mortality was determined. Morphological changes in the cuticle or body of cadavers, specifically changes in color or body turgor, were used to determine if the insect was dead. Each bioassay was repeated three times with four replicates for each treatment. Isolates showing the greatest potential in terms of pathogenicity were further tested by spraying a similar suspension on infested branches in a forest setting. Branches for treatment were selected based on visual inspection to ensure they contained at least 50 target insects/branches. Samples were taken before treatment, immediately after treatment and at several days after treatment and assessed for mortality. To further confirm the pathogenicity of the isolates, a random sample of dead insects (10 individuals/twig) were selected and plated to isolate fungi. Subsequently, pathological analyses and isolation of fungi was made, as described above.

## 3. Results and Discussion

Local epizootics were consistently found in correlation to the activity of fungi in each of the populations of elongate hemlock scale (EHS), hemlock woolly adelgid (HWA) and pear thrips investigated (Table 1 and Table 2). *Metarhiziopsis microspora* epizootics did not have a significant impact on insect mortality. This was also the case for the fungi *Paecilomyces marquandii* and *Isaria farinosa* (data not shown). The complex of *M. duriaei* & *C. fioriniae* caused EHS mortality rates from 27%–67%, depending on locality (Table 1). Natural mycoses in HWA were particularly found in association with the facultative entomopathogen *Phoma* spp. (Table 1). A wide range of mortality rates were observed depending on locality, but never bellow 20%. Subsequent experimental inoculation of HWA and EHS under laboratory and field conditions using the entomopathogens *B. bassiana*, *L. muscarium* and *Myriangium* sp., retrieved from EHS epizootics, resulted in higher insect mortality, up to 95% [12,31,32,33]. 

**Figure 1:**
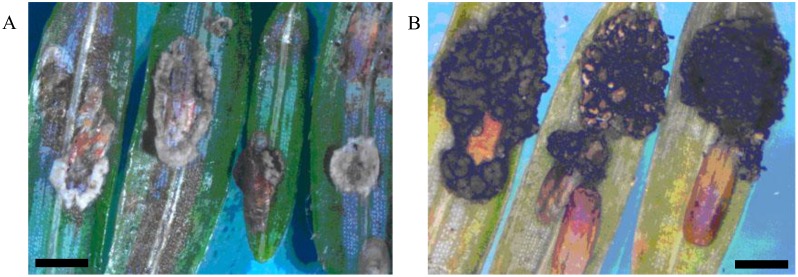
Elongate hemlock scale mycosis caused by *Myriangium duriaei* before (**A**) and after (**B**) sclerotization. (Bars = 0.5 mm)

**Figure 2:**
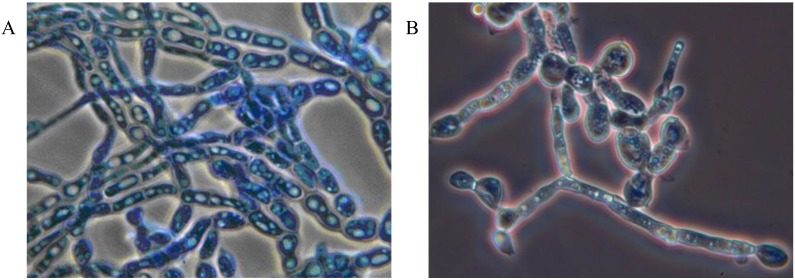
*Myriangium duriaei* in culture: mycelial mass (**A**), and formation of chlamydospores (**B**); cotton blue stain, objective ×100.

**Figure 3:**
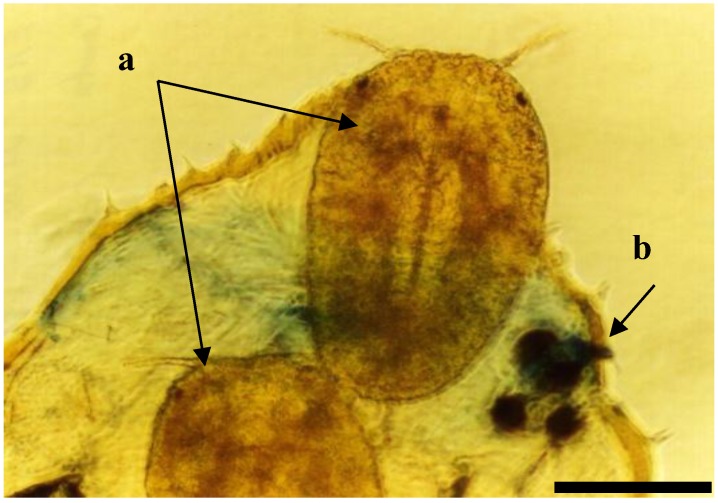
Elongate hemlock scale crawlers (a: 1st mobile instar) exiting from female body (b: outline of female body) containing sclerotia of *Myriangium duriaei*; phase contrast, objective ×10. (Bar = 0.5 mm)

**Figure 4:**
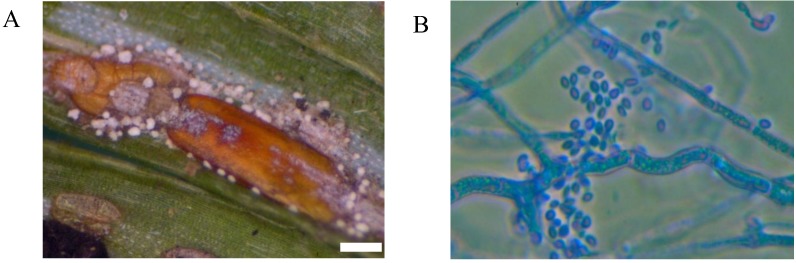
Elongate hemlock scale mycosis caused by the fungus *Metarhiziopsis microspora*: scales with sporodochia (**A**); mature conidia, cotton blue stain, objective ×100 (**B**). (Bar = 0.5 mm)

Mycoses on EHS, associated with *Myriangium duriaei*, were characterized by the formation of black sclerotized masses on the scale surface. Infected scales survived after initial infection. The early mycelial biomass on EHS was typically white and progressed to brown and finally black (Figure 1). *M. duriaei* recovered from infected elongate hemlock scale was characterized by slow growth on all media. Typical colonies had curled margins and umbonate elevations. The fungus developed as a massive septate vacuolated mycelium which formed chlamydospores (Figure 2); eventually, the mycelium dehydrated, hardened and accumulated melanin (Figure 1). Crawlers (*i.e.*, the 1st mobile instar) often did not reach adulthood and the ones that reached adulthood, or imagos, developed more slowly than the control populations in uncontaminated twigs. However, females with visible signs of fungal infection were still able to produce a new generation of crawlers (Figure 3). This first type of elongate hemlock scale mycosis was complex due to the recurrent presence of another fungus with *M. duriaei*, initially identified as the phytopathogen *Colletotrichum acutatum.* The fungus caused disease in experimental inoculations of elongate hemlock scale and Koch’s postulates were positive. The fungus was subsequently described as a new entomopathogenic subspecies, *C. acutatum* var*. fioriniae* [13], and re-assessed as a new species *C. fioriniae* (Marcelino & Gouli) R.G. Shivas & Y.P. Tan [34]. Biological properties of this fungus have been described [13,14,35]. The second type of mycosis is characterized by the formation of white rounded fungal structures around the scales (Figure 4). These formations are described as sporodochia, and represent an accumulation of short conidiophores with phialides. Fungal isolates obtained from scales with this second type of mycosis were initially identified based on DNA analysis as *Cordyceps* sp. [14] and subsequently described as a new species, *Metarhiziopsis microspora* [36].

**Table 1:** Natural occurring mycoses in elongate hemlock scale and hemlock wooly adelgid populations on 25–30 cm long twigs.

Mycoses in elongate hemlock scale (EHS)				
**Location**	**Total number of twig samples investigated **	**Fungi isolated from dead and diseased insects**	**Mortality, %**	**Date of collection**
Bayberry Lane ^a^	100	*Myriangium duriaei* & *Colletotrichum fioriniae*	35–67	Jun. 2006
Litchfield ^b^	100	*Myriangium duriaei* & *Colletotrichum fioriniae*	27–45	Jun. 2006
Mount Tom Forest Reserve ^c^	100	*Lecanicillium lecanii* & *Beauveria bassiana*	1–21–2	Jun. 2005
Valley Forge ^d^	200	*Metarhiziopsis microspora*	0.1–2.0	Jul. 2005
Mycoses in hemlock woolly adelgid **(HWA)**				
**Location**	**Total number of twig samples investigated **	**Fungi isolated from dead and diseased insects**	**Mortality, %**	**Date of collection**
Vaughan Woods State Park ^e^	47	*Phoma* sp.	71–92	Nov. 2011
Kittery ^e^	40	*Phoma* sp.	50–81	Nov. 2011
York ^e^	40	*Phoma* sp.	53–89	Nov. 2011
Harriman State Park ^a^	12	*Myriangium* sp*.*	22–40	Oct. 2011
Love Lane ^f^	5	*Phoma* sp.	50–80	Aug. 2011
King Brook ^f^	5	*Phoma* sp.	29–56	Aug. 2011
Brattleboro ^g^	4	*Phoma* sp. & *Myriangium* sp.	10–33	Sept. 2011
Amherst ^f^	5	*Phoma* sp.	20–58	Aug. 2011
Milford ^f^	5	*Phoma* sp.	30–60	Aug. 2011
Pelham ^f^	5	*Myriangium* sp*. & Metarhiziopsis microspora*	23–55	Aug. 2011
Newmarket ^f^	5	*Phoma* sp. & *Myriangium* sp.	20–56	Aug. 2011
Hudson ^f^	5	*Phoma* sp.	32–61	Aug. 2011
Berry Brook ^f^	5	*Phoma* sp.	23–84	Aug. 2011

a: New York State; b: Connecticut; c: Massachusetts; d: Pennsylvania; e: Maine; f: New Hampshire; g: Vermont.

Both types of mycoses were recorded concurrently in EHS populations. Mortality of insects, including mature insects and immature settlers, caused by *M. duriaei* in association with *C. fioriniae* was 100% in the center of the epizootic. Scales with signs of the second type of mycosis occurred sporadically together with the entomopathogenic fungi, *B. bassiana* and *Lecanicillium* spp. Numerous facultative fungal isolates of hemlock woolly adelgid were identified as species from the genera *Acremonium*, *Alternaria*, *Botrytis*, *Fusarium*, *Nectria*, *Rhinocladiella*, and *Scopulariopsis.* Fungi in the genera *Mucor*, *Penicillium* sp., *Aspergillus* sp., and some others, were usually present as ubiquitous opportunistic contaminants. Bioassays indicated that *C. fioriniae* was highly pathogenic to elongate hemlock scale. Mortality rates of >90% and >55% were obtained for crawlers and settlers, respectively [35].

**Figure 5:**
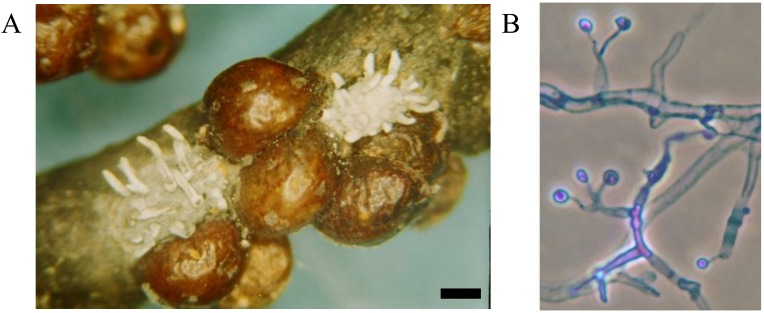
European fruit lecanium infected with the fungus *Hirsutella lecaniicola* (**A**); mature culture of fungus on nutrient medium, phase contrast, cotton blue stain, objective ×100 (**B**). (Bar = 0.5 mm)

Mycological and pathological analyses of the lecanium scale (LS) were conducted in plant material collected at different forest stands of Vermont, showing a profuse number of scale insects. Most twigs sampled contained scales with specific signs of mycoses typical of entomopathogenic fungi in the genus *Hirsutella*. Insects with signs of mycoses were used for isolation of entomopathogenic fungi from which 20 isolates of *Hirsutella lecaniicola* were obtained*.* The fungus forms a specific coremia on the surface of infected LS hosts, and, as a result, this insect pathogen can be easily detected (Figure 5). In addition, several other entomopathogenic fungi were isolated from lecanium scale including, *B. bassiana*, *L. muscarium*, *I. farinosa* and phytopathogenic fungi from the genus *Fusarium.*

**Figure 6:**
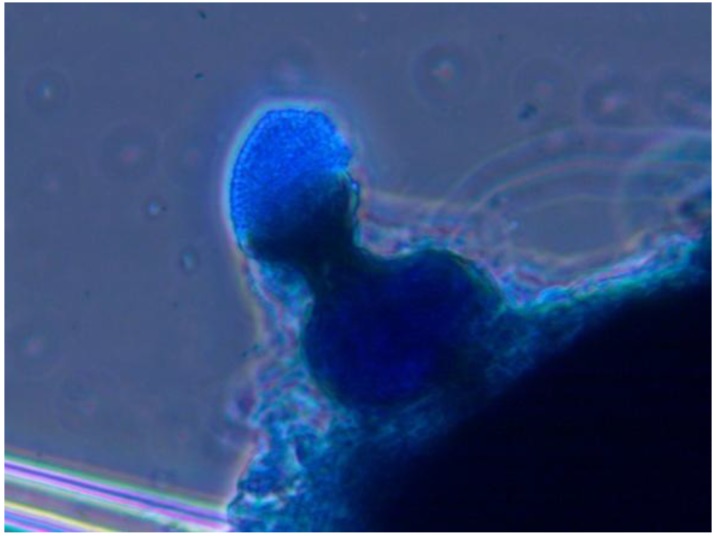
Pycnidia of the fungus *Phoma* sp. located on body of hemlock woolly adelgid, cotton blue stain, objective ×40.

Fungi associated with hemlock woolly adelgid (HWA) were collected throughout the infestation area covering the northeastern region of the USA. Several hundred fungal colonies were isolated from 1996 to 2012 [10,11,32]. Isolates included *B. bassiana* and unidentified species from the genera *Paecilomyces*, *Lecanicillium* and *Myriangium.* A significant number of the isolates included entomophilous and phytopathogenic fungi in the genera *Phoma*, *Fusarium*, *Cladosporium*, *Alternaria*, *Xylaria*, *Penicillium*, *Aspergillus*, *Mucor*, *Rhinocladiella*, *Exophiala*, *Scopulariopsis*, *Doratomyces* (=*Periconia*, *Stysanus*, *Cephalotrichum*) and *Diplocladiella.* A high level of HWA mortality, with characteristic signs of mycoses (*i.e.*, profuse mycelia on the surface of the insect), was recorded in areas around York, Maine and Harriman State Park, as well as New York state, from 2010–2012. Adult and immature infected HWA had a specific appearance described as a blackened cuticle and a flabby body surface, with woolly masses covering part of the mature females. The rate of infection gradually dropped as the season progressed and culminated in the death of the insect. Visual observations and examination of diseased and dead insects, using the methods described above, have not revealed a single agent causing fungal infection, but more commonly fungal propagules of numerous different species both on the body surfaces and in the body cavities. Commonly the insects were completely filled with mycelial masses and no other pathogenic structures. Often, mycelia formed clusters of deep melanization. A first subgroup of isolates from HWA formed gray colonies which eventually darkened. The colonies were relatively circular in appearance with umbonate elevations and undulate margins. Cultures produced a brown pigment which diffused into the medium and formed septate mycelial masses with a strong vacuolization. Mature cultures formed deeply melanized sclerotia. These cultures were preliminarily identified as *Myriangium* sp. This morphological identification was confirmed by DNA analysis [14]. Biocontrol research with *Myriangium* sp. is currently underway. Some of the adelgid cadavers from different geographical locations (Table 1) had a body surface covered with structures in the form of goblets filled with numerous spores (Figure 6). A detailed morphological analysis of these structures indicated that the fungus was related to the genus *Phoma. Phoma* sp. and *Myriangium* sp. have caused high mortality of HWA in 10–12 days in laboratory bioassays. *Phoma aspidioticola* has been reported to be an effective pathogen of the armored scale, *Aspidiotus destructor*, in India [37].

*Myriangium* sp. and *Phoma* sp. have different cultivation properties. The first is difficult to isolate in pure culture, because it grows slowly on different media, including PDA and SDAY, without production of any sexual morphological structures. Conversely, *Phoma* sp. grows quickly and forms specific fruiting structures. Based on the high rate of *Phoma* sp. recovered from infected populations of hemlock woolly adelgid, it is highly probable that the reason for the mass mortality of hemlock woolly adelgid is the activity of this pathogen. Together with the activity of *Myriangium* sp., *Phoma* sp. is therefore very likely to be involved in the epizootiological process affecting adelgids, however, currently the mechanism of infection and etiology of this disease is unknown. 

Damage caused by pear thrips has fluctuated from year to year. However, since outbreaks in 1985–1988, the population density has not been severe, and the activity of entomopathogenic fungi may be one of the reasons. Pear thrips remain in the soil for 10 mо of the, from mid-June to mid-April and entomopathogenic hyphomycetous fungi are closely associated with soil. Entomopathogenic fungi are known as important natural enemies of soil-dwelling arthropods [9,38]. A complex of entomopathogenic fungi has been reported to cause significant pear thrips mortality in forest litter and in the upper soil substrata [39]. We identified the fungi associated with pear thrips and other common soil arthropods such as fungal gnats, collembolans and mites (Table 2). A total of 1104 arthropods and 325 pear thrips were analyzed for mycological pathogens. A large portion of the arthropods were contaminated by fungi. The following genera were commonly isolated: *Aspergillus*, *Beauveria*, *Cladosporium Conidiobolus*, *Fusarium*, *Isaria*, *Lecanicillium*, *Mariannaea*, *Metarhizium*, *Mucor*, *Paecilomyces*, *Penicillium*, *Rhinocladiella*, and *Trichoderma*. The distribution of pear thrips within populations from different localities is listed in Table 2. Fungi in the genus *Lecanicillium* significantly prevailed in comparison with other fungi. The infection level of thrips with *Lecanicillium* fluctuated from 12.5%–51% in Vermont, 44.7%–45.4% in New Hampshire and >50% in New York. The overall level of infection of pear thrips by entomopathogenic fungi ranged from 58.1%–87.5% in Vermont, 68.2%–68.4% in New Hampshire, and 83%–100% in New York. For Vermont, the most commonly isolated fungi were in the genera *Lecanicillium* followed by those in the genera *Beauveria*, *Metarhizium*, *Isaria* and *Mariannaea*. Samples from New York contained a small number of pear thrips infected with *Lecanicillium* spp. and *Isaria* spp. (Table 2). The rate of fungal infection among other arthropods with *Lecanicillium* spp. fluctuated between 22.8%–50% in Vermont, 16.1%–50.5% in New Hampshire and 11.4%–25.45% in New York. Fungi from the genus *Lecanicillium* appeared to be an important limiting factor of pear thrips population growth [1,10,39]. Pathogen loads in thrips populations could be used to monitor their health and provide a rapid and perhaps accurate assessment of the ecological status of this pest. 

From our research with entomopathogenic fungi in northeastern forests >200 isolates in the genera *Beauveria*, *Lecanicillium*, *Metarhizium*, *Isaria*, *Paecilomyces*, *Mariannaea*, *Hirsutella*, *Fusarium* and *Rhinocladiella* were deposited in the USDA-ARS Collection of Entomopathogenic Fungal Cultures and the Collection of Microorganisms Useful for Plant Protection at the Entomology Research Laboratory (University of Vermont, Burlington, VT, USA). Fungal cultures were divided into three groups: (a) specialized entomopathogenic species, *Mariannaea* sp. *Myriangium duriaei* Mont. & Berk., *Myriangium* sp., *Hirsutella lecaniicola* (Jaap) Petch, *Hirsutella* sp., *Metarhiziopsis microspora*, *Beauveria bassiana* (Balsamo-Crivelli) Vuillemin, *Lecanicillium muscarium* (Petch) Zare & WGams*, L. psalliotae* (Treschow) Zare & Gams, *Lecanicillum* sp*. Paecilomyces marquandii* (Massee) Hughes, *Isaria farinosa* (Holmsk.) Fries, *Isaria* sp*.* and *Colletotrichum fioriniae* (Marcelino & Gouli) R.G. Shivas & Y.P.; (b) facultative entomopathogens, *Rhinocladiella* sp., *Nectria* sp., *Botrytis* sp., *Fusarium* sp., *Phyalophora* sp., *Phoma* sp.; (c) ubiquitous opportunistic contaminants, *Penicillium* sp., *Cladosporium* sp., *Scopulariopsis* sp., and *Aspergillus* sp.

**Table 2:** Entomopathogenic and entomophilous fungi isolated from pear thrips and associated soil arthropods (mites, collembolans and fungus gnats) on forest soil during the period of August–October 2011.

Sample sites	Number of sampled arthropods		Number of arthropods infected with entomopathogenic fungi									
	Thrips	Others	*Lecanicillium* spp.		*Beauveria bassiana*		*Metarhizium* * anisopliae*		*Isaria* spp.		*Mariannaea* * spp.*	
			Thrips	Others	Thrip	Other	Thrips	Other	Thrip	Other	Thrip	Other
Bakersfield ^a^	44	76	16	31	4	4	3	2	2	7	0	0
Richford ^a^	8	88	3	25	0	5	0	0	3	5	1	1
Derby ^a^	8	20	1	8	2	2	0	0	7	23	1	0
Randolph ^a^	47	79	24	18	2	0	0	0	7	5	1	1
Underhill ^a^	14	331	57	130	5	3	2	2	18	3	0	0
Westfield ^a^	4	40	1	20	1	1	0	0	0	7	0	0
Altona ^b^	4	71	0	14	0	0	0	0	3	7	0	0
Rupert ^b^	6	110	2	28	0	5	0	0	2	23	1	1
Sharon Springs ^b^	3	88	1	10	0	3	0	0	2	5	0	0
Langdon ^c^	22	130	10	21	0	0	0	0	2	5	3	3
Oxford ^c^	38	91	17	46	5	3	0	0	3	3	1	1

a: Vermont; b: New York State; c: New Hampshire state.

## 4. Conclusions

Based on the data reported herein, we conclude that many arthropods in forest soil are infected with different species of entomopathogenic and entomophilous fungi. It is hypothesized that the isolated fungi found play a significant role as biotic factors regulating arthropod populations. Most of the fungi recovered in the forest were ecologically associated with the soil and thus had a limited capability for persisting in the tree canopy, where populations of insect pests may reside. Therefore, a single application of *B. bassiana* and *L. muscarium*, isolated from soil-dwelling insects, may not provide long-term suppression of pests in the tree canopy. Insect populations were able to re-establish after the winter, as previously observed following chemical treatments. However, this research indicates that use of entomopathogenic fungi, as an alternative biocontrol strategy to counteract the economic impact of pests in northeastern forests, is able to generate mortality rates identical or superior to those from agricultural chemicals without the associated costs and long-term deleterious impacts in the environment. Additional research is needed to develop the full potential of entomopathogenic fungi for integrated pest management in forests.
